# Elucidation of SIRT-1/PGC-1α-associated mitochondrial dysfunction and autophagy in nonalcoholic fatty liver disease

**DOI:** 10.1186/s12944-021-01461-5

**Published:** 2021-04-26

**Authors:** Yan Jiang, Duankai Chen, Qiming Gong, Qunqing Xu, Dong Pan, Feiyan Lu, Qianli Tang

**Affiliations:** 1 Medical College of Guangxi University, Nanning, 530004 Guangxi China; 2 YouJiang Medical University for Nationalities, Baise, 533000 Guangxi China

**Keywords:** Sirtuin 1, Peroxisome proliferator-activated receptor-gamma coactivator -1a, Mitochondrial physiology, Mitochondrial autophagy, Mitochondrial dysfunction; Lipid autophagy, Nonalcoholic fatty liver disease

## Abstract

**Background:**

Nonalcoholic fatty liver disease (NAFLD) can lead to chronic liver diseases associated with mitochondrial damages. However, the exact mechanisms involved in the etiology of the disease are not clear.

**Methods:**

To gain new insights, the changes affecting sirtuin 1 (SIRT-1) during liver fat accumulation was investigated in a NAFLD mouse model. In addition, the in vitro research investigated the regulation operated by SIRT-1 on mitochondrial structures, biogenesis, functions, and autophagy.

**Results:**

In mice NAFLD, high-fat-diet (HFD) increased body weight gain, upregulated serum total cholesterol, triglycerides, aspartate aminotransferase, alanine aminotransferase, blood glucose, insulin levels, and liver malondialdehyde, and decreased liver superoxide dismutase activity. In liver, the levels of SIRT-1 and peroxisome proliferator-activated receptor-gamma coactivator -1α (PGC-1α) decreased. The expression of peroxisome proliferator-activated receptor-α and Beclin-1 proteins was also reduced, while p62/SQSTM1 expression increased. These results demonstrated SIRT-1 impairment in mouse NAFLD. In a well-established NAFLD cell model, exposure of the HepG2 hepatocyte cell line to oleic acid (OA) for 48 h caused viability reduction, apoptosis, lipid accumulation, and reactive oxygen species production. Disturbance of SIRT-1 expression affected mitochondria. Pre-treatment with Tenovin-6, a SIRT-1 inhibitor, aggravated the effect of OA on hepG2, while this effect was reversed by CAY10602, a SIRT-1 activator. Further investigation demonstrated that SIRT-1 activity was involved in mitochondrial biogenesis through PGC-1α and participated to the balance of autophagy regulatory proteins.

**Conclusion:**

In conclusion, in high-fat conditions, SIRT-1 regulates multiple cellular properties by influencing on mitochondrial physiology and lipid autophagy via the PGC-1α pathway. The SIRT-1/PGC-1α pathway could be targeted to develop new NAFLD therapeutic strategies.

## Background

Nonalcoholic fatty liver disease (NAFLD) is the most common chronic liver disease, with a severity ranging from simple steatosis, steatohepatitis, fibrosis, to cirrhosis, and leads to all-cause and liver-related mortality [1]. So far, the working model of NAFLD proposes the “two-hit hypothesis”. Insulin resistance, which increases diet intake and hepatic lipogenesis, may cause the accumulation of triglycerides (TG) and free fatty acids in the liver. However, lipid peroxidation, mitochondrial dysfunction, and inflammation may eventually cause hepatocyte damages and liver fibrosis. Recently, some experts in the field proposed that in essence, NAFLD may be a mitochondrial disease [1].

It is well established that a high-fat diet (HFD) causes an abnormal accumulation of TG and imbalances mitochondrial function in the liver [2–5]. In progressive liver diseases, mitochondrial dysfunction produces excessive reactive oxygen species (ROS) and cytokines, which leads to hepatic inflammation and injury. Furthermore, mitochondrial dysfunction disturbs fat homeostasis in hepatic cells and eventually causes lipid accumulation, which leads to lipotoxicity [6]. It has recently been suggested that imbalanced activity of the transcription factors sterol regulatory element binding protein-1c (SREBP-1c) and peroxisome proliferator-activated receptor-α (PPAR-α) causes mitochondrial dysfunction, which in turn induces liver steatosis [7, 8]. In addition, HFD (such as palmitic acid) intake is a high-risk inducer of SREBP-1c/PPAR-α ratio imbalance [9].

Sirtuin 1 (SIRT-1), a master metabolic mediator, is a NAD-dependent protein deacetylase [10] involved in the control of lifespan extension and lipid metabolism, including the processes of fatty acid synthesis, oxidation, and adipocyte generation [11, 12]. SIRT-1 activation has a beneficial effect against NAFLD through inhibiting lipogenesis, by phosphorylation of SREBP-1c [11]. Studies have demonstrated that SIRT-1 reduces oxidative stress [13, 14]. In HFD-induced obese mice, the expression of SIRT-1 is inhibited, resulting in liver metabolic damages [15]. Yet, how SIRT-1 regulates mitochondrial biology in high fat-induced livers remains poorly understood.

In the present study, the mechanisms underlying the effects of HFD on SIRT-1 in liver were investigated in vivo. The present study also tested if SIRT-1 could modulate mitochondrial physiology, and investigated the mechanisms involving SIRT-1 regulation in oleic acid (OA)-treated hepatocytes in vitro.

## Materials and methods

### Animal experiments

#### Animal models

Four- to five-week-old C57BL/6 mice were purchased from Changsha Tianqin Biotechnology Co., Ltd. (Changsha, China). The mice were housed in a specified constant environment and fed a normal diet for 1 week. Then, the six-week-old mice were randomly distributed between the control group (CON) and the HFD group, each group gathering eight animals. The mice were provided food and drinking water ad libitum. The mice in the CON group continued being fed an ordinary diet, while the mice in the HFD group were fed a HFD for 8 weeks. The ordinary diet was composed of (expressed as % total calories) 65% carbohydrates, 11% fat, 24% proteins, with a total caloric value of 3.84 kcal/g, while HFD was composed of 18% carbohydrates, 62% fat, 24% proteins, with a total caloric value of 5.49 kcal/g. The specific components of the HFD were diversified and were produced by Jiangsu Synergetic Pharmaceutical Bioengineering Co., Ltd. (Jiangsu, China).

#### Collection and biochemical analysis of serum and liver tissue

After 8 weeks of normal or HFD food intake, the 14-week-old mice were fasted for 12 h and anesthetized using 7% chloral hydrate. Blood samples were withdrawn from the retro-orbital cavity. The serum was prepared by centrifugation at 5000×g for 10 min at 4 °C and then stored at − 80 °C for biochemical analyses. The liver was harvested, rinsed in PBS, and wiped with filter paper. Part of the liver was fixed in formalin at 4 °C, while the remaining part was stored at − 80 °C for subsequent analyses, after being quickly frozen in liquid nitrogen. The concentrations of total cholesterol (TC), TG, aspartate aminotransferase (AST), alanine aminotransferase (ALT) and blood glucose in serum were detected using biochemical reagent kits (Nanjing Jiancheng Bioengineering Institute, Nanjing, China). Biochemical assays were also performed to probe SOD activity and MAD level in liver tissues. Serum insulin was measured by the enzyme-linked immunosorbent assay (ELISA) (Beyotime, Nanjing, China) methods using a TriStar LB941 system (Berthold, Wildbad, Germany).

#### Histopathological examination

The right lobe of the livers were collected for histopathological examination. The liver tissues fixed in formalin were cut into 5-μm sections and stained with hematoxylin and eosin dyes. The livers sections frozen in liquid nitrogen were cut into 5-μm using a frozen slicer (Thermo, Waltham, USA). The sections were stained with oil red O solution and hematoxylin. Histopathological structures were observed under a light microscope (BX53, Olympus, Tokyo, Japan).

#### RNA extraction and analysis

Total RNA was extracted using TRIzol reagent (CoWin Biosciences, Beijng, China) and transcribed into cDNA with a Reverse Transcription Kit (Thermo Fisher Scientific, Waltham, USA), according to the manufactures’ instructions. Then, real-time quantitative PCR (RT-qPCR) was performed using SYBR Green Master Mix (CoWin Biosciences, Beijng, China). The reaction conditions were: pre-denaturation at 95 °C for 10 min, then 40 cycles of denaturation at 95 °C for 15 s and annealing at 60 °C for 30 s. The level of GAPDH cDNA (F: 5′-CCTCGTCCCGTAGACAAAATG-3′, R: 5′-TGAGGTCAATGAAGGGGTCGT-3′) was used as internal reference to quantify the level of SIRT-1 (F: 5′-TTCAGAACCACCAAAGCGGA-3′, R: 5′-TCCCACAGGAGACAGAAACCC-3′) and PGC-1ɑ (F: 5′-CGAGAAGCGGGAGTCTGAAAG-3′, R: 5′-GAGCAGCGAAAGCGTCACA-3′) cDNA. The relative mRNA expression of SIRT-1 and PGC-1ɑ was calculated using the 2^-ΔΔCt^ formula. All results were normalized to the GAPDH expression level.

### Cell experiments

#### Culture conditions for the HepG2 hepatocyte cell line

The HepG2 cell line was purchased from the Cell Bank of China Academy of Sciences. Cells were maintained in Dulbecco’s Modified Eagle’s Medium (DMEM, 4.5 g/L glucose, Gibco, Carlsbad, CA, USA) supplemented with 8.0% fetal bovine serum, penicillin (100 U/mL) and streptomycin (100 μg/mL).

#### Cell treatment

Cells were treated with OA (Sigma-Aldrich. St. Louis, MO, USA, catalogue #: O1008), and SIRT-1 induction was monitored by comparing levels in unpretreated and pretreated cultures. Briefly, after inoculation, HepG2 cells were grown for 24 h and treated with 1.5 mM OA for 48 h to induce NAFLD in cell model. The SIRT-1 inhibitor Tenovin-6, referred to as T6 (MCE, New Jersey, USA, catalogue #: HY-15510), was used at a concentration of 2 μM. The SIRT-1 activator CAY-10602, referred to as CAY (MCE, New Jersey, USA, catalogue #: HY-104073), was used at a concentration of 20 μM. Cells were incubated with T6 or CAY for 2 h, and subsequently stimulated with OA for 48 h. The drug-containing medium contained 12.0% of serum.

#### Cell viability assay

After 48 h of treatment, the viability of the HepG2 cells was assessed with the CCK-8 kit (ABMole, Houston, USA, catalogue #: M4839). In brief, 10 μL of CCK-8 solution was added to each well and incubated for 1 h. The optical density (OD) was measured at 450 nm using the TriStar LB941 microplate reader.

#### Oil red O staining

Oil red O was used to stain intracellular lipids. The cells were fixed with 10% neutral formaldehyde for 20 min after removing the cell culture medium. Then, the cells were stained with 5 mg/mL of oil red O solution for 30 min at room temperature. The cells were washed with PBS, and then stained with hematoxylin for 1 min. After rinsing, the cells were observed and imaged under a light microscope (BX53, Olympus, Tokyo, Japan).

#### Flow cytometric analysis

The apoptosis rate in HepG2 cells was measured using the Annexin V-fluorescein Isothiocyanate/ propidium iodide (Annexin V-FITC/PI) apoptosis kit. Cells were rinsed with PBS, digested, and centrifuged. The cell density was adjusted to 1 × 10^6^ /mL. Then, the cells were incubated with Annexin V-FITC and PI. Flow cytometric data were collected on a flow cytometer from BD Biosciences (FACSVerse, BD Biosciences, Franklin, USA) and analyzed using FlowJo software (v10.0; BD Biosciences, Franklin, USA).

#### Immunofluorescence analysis

Cells were seeded into glass-bottom cell culture dishes. The cells were co-transfected with Ad-GFP-LC3 and Ad-HBmTur-Mito (Hanbio, Shanghai, China, catalogue #: HBAD-1006, 1014). Following stimulation, the localization of mitochondrial autophagy (MA) in cells was monitored under confocal laser scanning microscope (FV3000, Olympus, Tokyo, Japan). In another experiment, after removing the cell culture medium, the cells were incubated with a ROS fluorescent probe from an assay kit for 45 min at 37 °C. Finally, the cells were washed and analyzed with the confocal laser scanning microscope.

#### Transmission electron microscopy analysis

After treatment as described above, the culture medium was discarded, and 2.5% glutaraldehyde was quickly added to fix the cells. Cells were collected, centrifuged, re-fixed, dehydrated, infiltrated, and embedded. Finally, the ultrathin sections were stained with uranium and lead double solution and analyzed using a transmission electron microscope (HT7700, HITACHI, Tokyo, Japan).

#### Western blotting analysis

The total proteins were extracted on ice from the cells and liver tissues by ultrasonic lysis in RIPA buffer containing 1 mM PMSF and 1% phosphatase inhibitor cocktail. The lysates were centrifuged at 12000 ×g for 15 min at 4 °C. The protein concentration in the supernatant of the lysates was determined with a BCA protein quantitative kit. Next, the samples were normalized, mixed with loading buffer, and denatured by heating at 95 °C for 5 min. Aliquots of 50 mg of proteins were loaded onto 8, 10, or 12.5% SDS-PAGE gels. The proteins were transferred onto 0.45 μm PVDF membranes. The membranes were blocked and incubated overnight at 4 °C with primary monoclonal antibodies against SIRT-1, PGC-1α, Cytochrome C oxidase IV (COX-IV), PPAR-ɑ, Mitochondrial fission factor (MFF), Mitofusin-1 (MFN1), p62/SQSTM1 (p62), Light chain 3B (LC3B), Beclin-1, and GAPDH (all from Affinity Biosciences, OH, USA). The membranes were washed with TBST and incubated with secondary antibodies at room temperature for 40 min, and after washing them five times, an enhanced chemiluminescence kit was used to detect the proteins on the membranes. The bands were quantified using the ImageJ system (v1.8.0, NIH, Bethesda, USA).

#### Statistical analysis

The data were analyzed using IBM SPSS statistics 23.0 software. Paired two-tailed *t-*test and one-way analysis of variance (ANOVA) were used to assess the significance of differences observed between groups. *P* < 0.05 was considered statistically significant. All data are expressed as the mean ± standard deviation (M ± SD).

## Results

### HFD exacerbates obesity-related parameters in mice

The mice fed HFD had a significant increase of total body weight, net weight gain and liver weight compared with the mice of the control group (Table 1A). Energy intake was increased in HFD mice compared to the controls (Table 1B), concomitantly with marked increase of serum TC, TG, AST, ALT, blood glucose, insulin levels and liver MDA content, and reduction of SOD activity (Table 1C-D).

**Table 1:** General and hepatic parameters in control mice (CON) and high-fat-diet (HFD) mice

	CON	HFD
A. General and hepatic parameters		
Initial body weight (g)	14.49 ± 0.55	14.56 ± 0.41
Final body weight (g)	29.38 ± 5.58	43.45 ± 5.22^**^
Net weight gain (g)	14.89 ± 5.60	28.89 ± 4.97^**^
Liver weight (g)	1.20 ± 0.16	2.08 ± 0.47^**^
B. Food and Energy intake		
Dietary intake (g/day)	3.64 ± 0.37	3.66 ± 0.41
Energy intake (kcal/day)	14.64 ± 0.68	18.29 ± 1.83^**^
C. Serum parameters		
TC (mM)	1.39 ± 0.16	2.98 ± 0.38^**^
TG (mM)	1.64 ± 0.23	3.95 ± 0.54^**^
AST (U/L)	148.14 ± 13.40	180.24 ± 10.08^**^
ALT (U/L)	64.14 ± 5.56	74.84 ± 9.05^*^
Blood glucose (mg/dL)	122.68 ± 12.30	174.83 ± 19.31^**^
Insulin (μU/mL)	5.90 ± 1.95	26.61 ± 3.67^**^
D. Enzyme activity in liver		
MDA (nmol/g)	37.40 ± 7.10	74.92 ± 9.59^**^
SOD (U/g)	78.92 ± 7.75	46.32 ± 6.50^**^

Values are presented as mean ± SD (*n* = 6). Data were analyzed by one-way analysis ANOVA. ^*^, ^**^ represents significance from the control group at *P <* 0.05, *P <* 0.01

### HFD leads to aggregated lipids synthesis, decreased expression of SIRT-1 and PGC-1α, and alteration of autophagy-related proteins

**Fig. 1:**
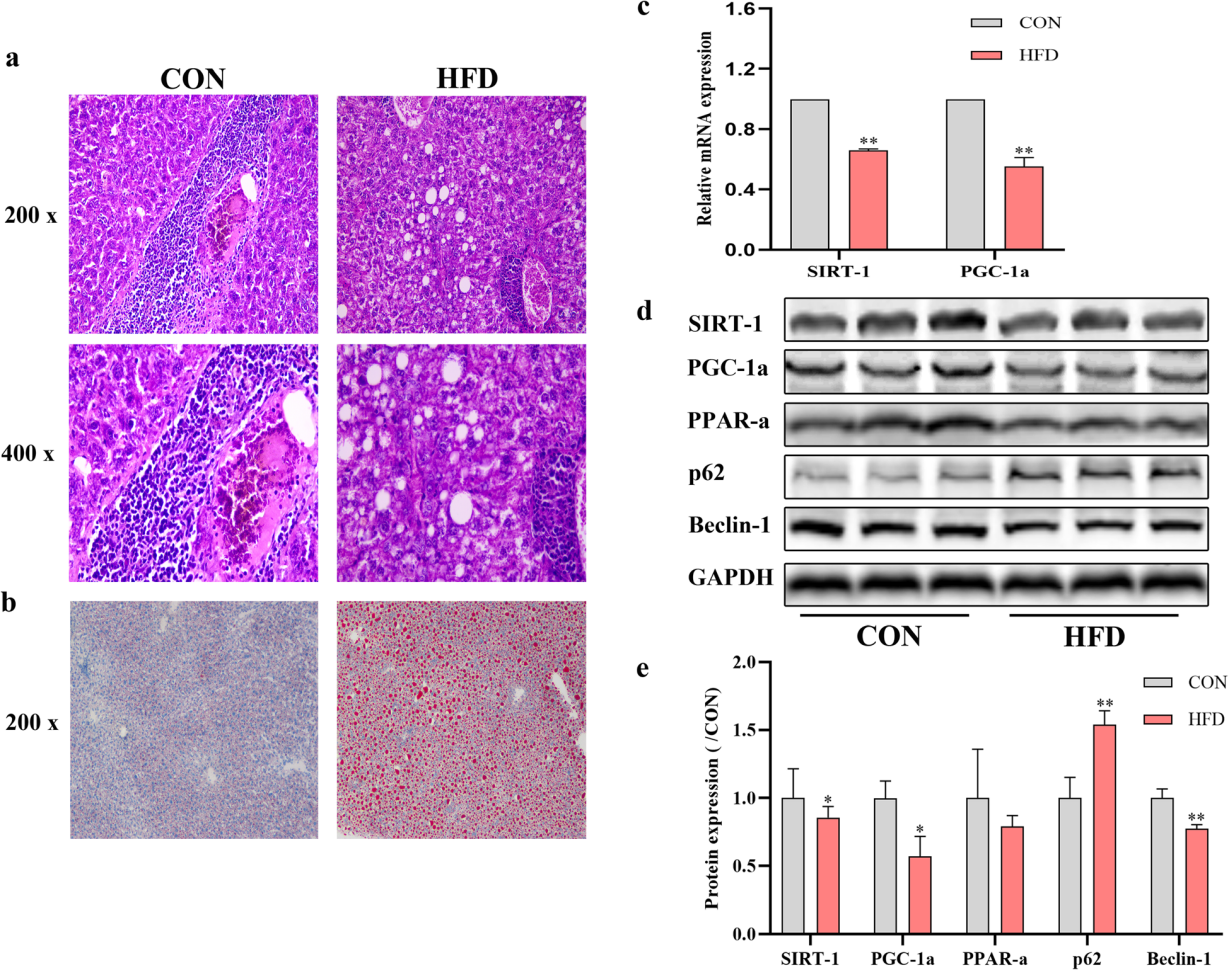
Effect of HFD for 8 weeks on liver steatosis, SIRT-1 and PGC-1ɑ mRNA level and protein expression of SIRT-1, PGC-1ɑ, PPAR-ɑ, p62, Beclin-1. **a** The liver pathology of mice in the two groups. **b** Lipid deposition in liver tissues. **c** The mRNA expression level of SIRT-1 and PGC-1ɑ. **d** The protein expression of SIRT-1, PGC-1a, PPAR-ɑ, p62 and Beclin-1 was analyzed and quantified. *P < 0.05, **P < 0.01, vs. CON. Original magnification: × 200, × 400

As shown in Fig. 1, large amounts of fat forming vacuoles were observed in the livers of mice fed HFD (Fig. 1a). The oil red O staining also confirmed that lipid droplets accumulated in the livers of the mice from the HFD group (Fig. 1b). SIRT-1 and PGC-1α expression was quantified in liver tissues by RT-qPCR and western blotting analysis. After 8 weeks of HFD regimen, liver SIRT-1 and PGC-1α expression declined (Fig. 1c-e). The expression of PPAR-ɑ and Beclin-1 proteins also decreased, while p62 protein expression increased (Fig. 1d-e).

### Effect of SIRT-1 on OA-induced cell viability in hepG2 cells

**Fig. 2:**
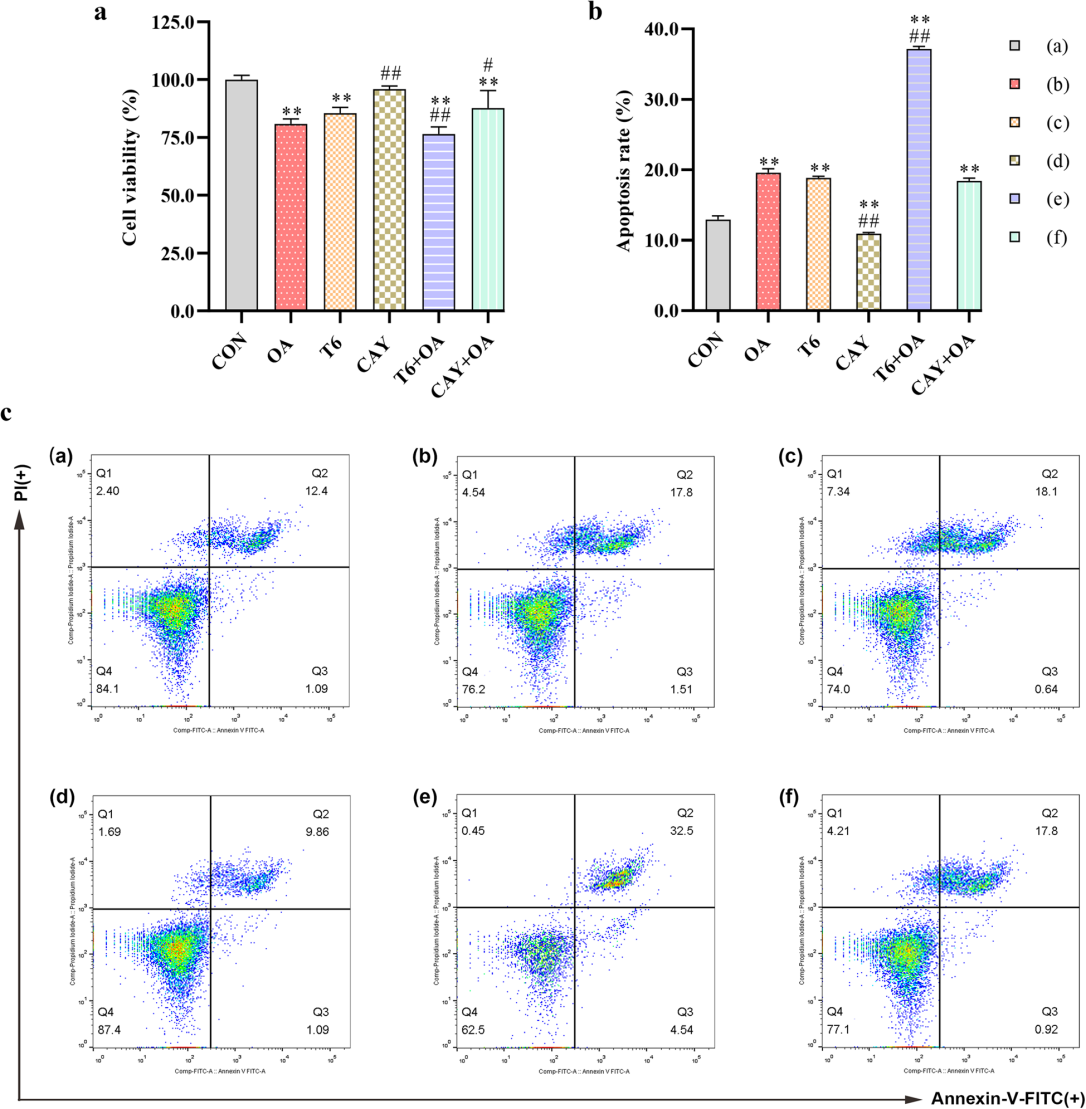
SIRT-1 regulates HepG2 cell viability and apoptosis crosstalk with OA. The cells were pretreated with T6 (2 μM) or CAY (20 μM) 2 h, followed incubated with OA (1.5 mM) for 48 h. **a** The cell viability was estimated using CCK-8 kit. **b**, **c** Flow Cytometry analysis of apoptosis and apoptosis rate quantitative analysis in cells. ^*^P < 0.05, ^**^P < 0.01, vs. CON group; ^#^*P* < 0.05, ^##^*P* < 0.01, vs. OA group

To determine treatment toxicity, HepG2 cell viability was assessed by CCK-8 assay. Compared with untreated CON cultures, OA and T6 treatments decreased cell viability significantly. The combination of the two substances aggravated the cell loss. CAY had no effect on cell viability and rescued the low viability induced by OA (Fig. 2a).

### Effect of SIRT-1 on OA-induced cell apoptosis in hepG2 cells

Cell death includes apoptosis and necrosis. The apoptosis provoked by the different treatments of HepG2 cells was assessed by flow cytometry. Upon OA and T6 treatments, apoptosis increased and was even more drastic in the T6-pretreated T6 + OA cultures. CAY reduced cell apoptosis and rescued OA-induced apoptosis (Fig. 2b-c).

### Effect of SIRT-1 on OA-induced intracellular lipid in hepG2 cells

**Fig. 3:**
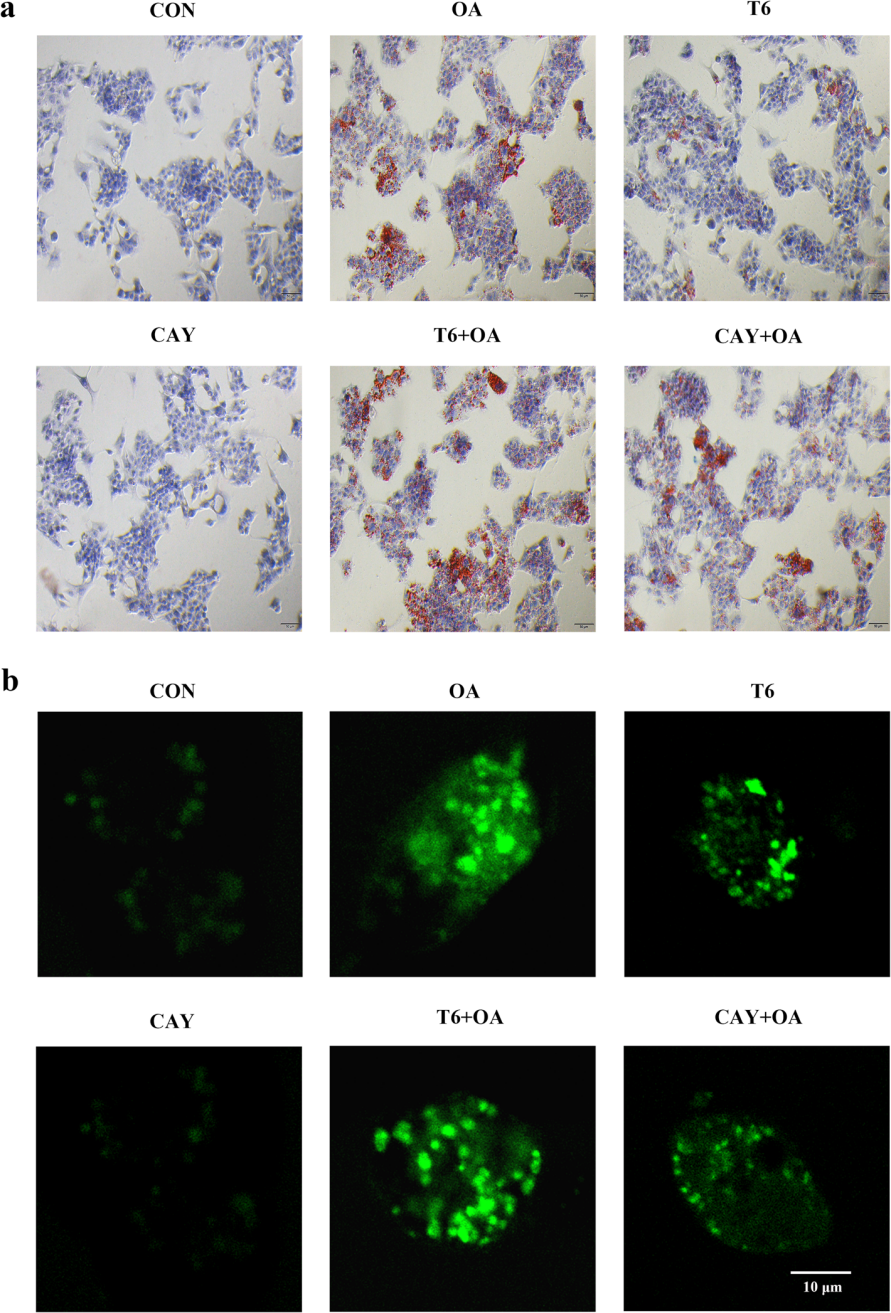
SIRT-1 regulates HepG2 intracellular lipid and ROS crosstalk with OA. The cells were pretreated with T6 (2 μM) or CAY (20 μM) 2 h, followed incubated with OA (1.5 mM) for 48 h. **a** The cells were stained oil red O. **b** Immunofluorescence detected intracellular ROS. Scale bars are 50 μm and 10 μm respectively

The level of intracellular lipids was revealed by oil red O staining. OA or T6 treatment led to lipid accumulation manifesting as intracellular red oil droplets. This phenomenon was more pronounced with the combination of OA and T6. CAY treatment attenuated the lipid accumulation induced by OA. The oil droplets were not clearly visible in the CON and CAY groups (Fig. 3a).

### Effect of SIRT-1 on OA-induced ROS in hepG2 cells

For the evaluation of mitochondrial damage, intracellular ROS was visualized by fluorescence. Mitochondrial ROS was significantly increased by OA and T6, and further enhanced by a combined treatment. In contrast, CAY did not impacted ROS whereas it alleviated OA-induced ROS production (Fig. 3b).

### Effect of SIRT-1 on OA-induced mitochondrial autophagy in hepG2 cells

**Fig. 4:**
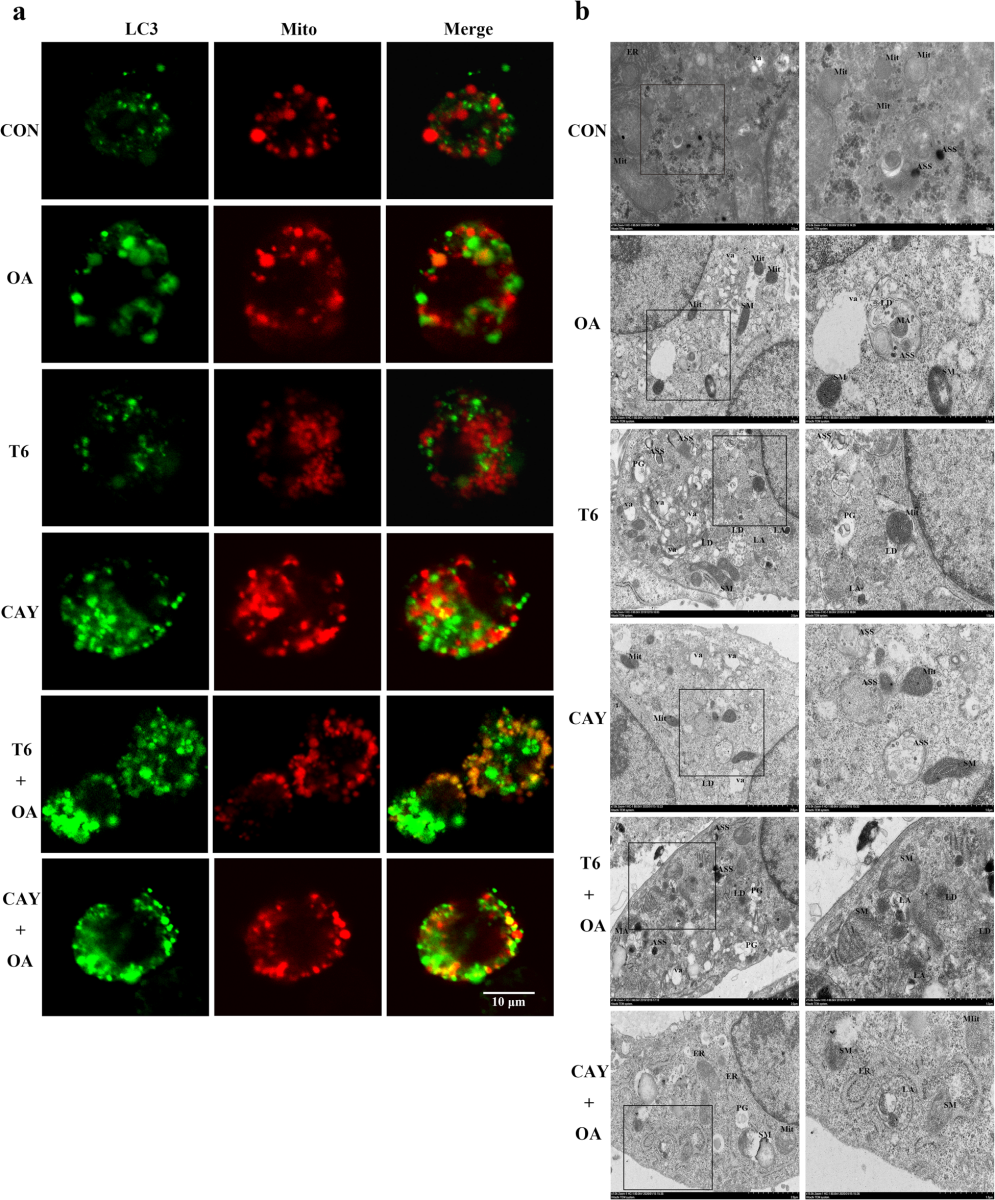
SIRT-1 involved in regulation of mitochondrial autophagy and lipophages in HepG2 cells. The cells were pretreated with T6 (2 μM) or CAY (20 μM) 2 h, followed incubated with OA (1.5 mM) for 48 h. **a** Mitochondrial autophagosome was determined. Red: mitochondria; green: LC3; yellow/ orange: merge. Scale bars are 10 μm respectively. **b** Representative electron micrographs of cells. Mit: mitochondria; SM: swollen and ruptured mitochondria; ER: endoplasmic reticulum; ASS: autophagolysosome; PG: phagophore; va: vacuole; LD: lipid drops; LA: lipid component of autolysosome. Scalar bar = 2.0 μm and 1.0 μm

Mitochondrial autophagy was identified by staining for the autophagy protein LC3 (in green, Fig. 4a) and mitochondria (in red, Fig. 4a). Mitochondrial autophagy (in yellow/ orange, Fig. 4a) was detected in OA, CAY, T6 + OA, and CAY + OA treated cells using the confocal laser scanning microscope. T6 pretreatment severely aggravated the increase of mitochondrial autophagy provoked by OA, while CAY pretreatment appropriately increased the effect of OA (Fig. 4a).

### SIRT-1 mediated the effects of OA on the substructures of hepG2 cells

HepG2 hepatocyte substructures were analyzed by transmission electron microscopy. At different stages and according to membrane enclosing cytoplasm or damaged organelles, double-membrane structures represent autophagosomes, and single-membrane structures are autolysosomes [16].

As it can be seen from the micrographs, OA- and T6-treated cells have high numbers of vacuole (va), lipid drops (LD), swollen and ruptured mitochondria (SM) and autolysosomes (ASS). Lipid drops are surrounded by autolysosomes and undergo lipophagy (LA, Fig. 4b). Several autolysosomes are observed with the highest numbers being in T6 + OA cells. Moreover, these structures are more frequent in T6-treated cells, compared to other cultures (Fig. 4b).

**Fig. 5:**
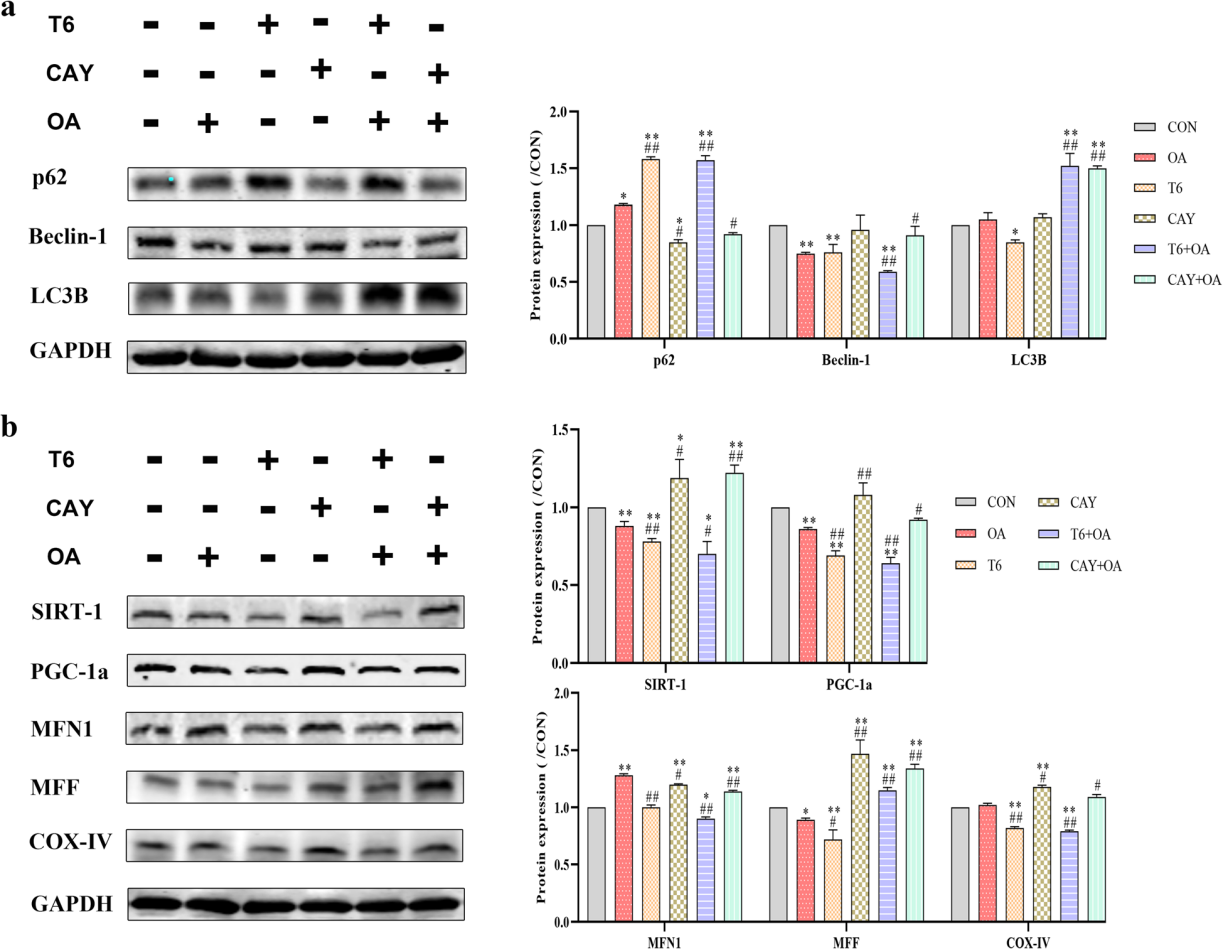
Effect of intervene in SIRT-1 and combine with OA on p62, Beclin-1, LC3B, SIRT-1, PGC-1a, MFN1, MFF and COX-IV protein expression in HepG2 cells. HepG2 cells were treated with 1.5 mM OA for 48 h, following T6 (2 μM) or CAY (20 μM) 2 h. **a** The p62, Beclin-1 and LC3B protein expression was detected by Western blotting. **b** The same method was used to detect the SIRT-1, PGC-1a, MFN1, MFF and COX-IV protein expression. The bands were normalized using GAPDH. The images were quantified with ImageJ. Data are presented as the mean ± SD; *P < 0.05, **P < 0.01 compared with CON group; ^#^*P* < 0.05, ^##^*P* < 0.01 compared with OA group

Western blotting analysis showed that OA decreased Beclin-1 protein expression to levels similar to T6. CAY treatment reversed OA effect. Meanwhile, other autophagy-related protein markers were analyzed in the cells receiving different treatments. The level of p62 was increased in the cells receiving the OA or T6 different treatments but was the highest in T6 + OA treatmed cells. Furthermore, LC3B protein level increased abnormally after stimulated by CAY or T6 pretreatment in the OA-induced cells (Fig. 5a).

### Effect of SIRT-1 induction upon OA treatment on hepG2 cells mitochondrial biogenesis

To assess mitochondrial biogenesis, the effects of SIRT inhibitor and activator on mitochondrial protein expression were verified (Fig. 5b). Besides, the protein PGC-1α was quantified as measurement of mitochondrial biogenesis. The results showed that PGC-1α level decreased significantly after OA, T6 and combined treatment. The SIRT-1 activator CAY significantly promoted PGC-1α expression compared to OA and T6. To further monitor the changes in mitochondrial biogenesis, the expressions of specific mitochondrial regulatory proteins was quantified by western blotting analysis. The results indicated that OA induced the mitochondrial fusion protein MFN1 but inhibited the expression of the fission protein MFF. T6 inhibited MFF protein. However, CAY promoted both mitochondrial fusion and fission, alleviated OA-promoted fusion, and rescued the inhibition operated by OA on mitochondrial fission. T6 pretreatment inhibited OA-induced mitochondrial fusion, while increasing OA-induced fission. Moreover, CAY and CAY + OA upregulated the expression of the mitochondrial respiratory protein COX-IV (Fig. 5b).

## Discussion

SIRT-1 is a sensor of cell metabolic reactions such as stress, starvation, and caloric restriction. Target genes of mitochondrial biogenesis can be regulated by SIRT-1 to maintain energy and metabolism stability [17]. New studies have regarded SIRT-1 as an important target for diseases associated with mitochondrial dysfunction [18]. In this experiments in mice, long-term high-fat regimen reduced the activity of SIRT-1 protein, and simultaneously, adverse changes occurred in liver structure and metabolites, such as increased levels of TG, TC, AST, ALT and MDA, and reduction of SOD activity. In addition, the interaction between SIRT-1 and autophagy responses in mitochondria under lipid accumulation process has not been explored. Further, this study deepened the insights on the effect of SIRT-1 on mitochondrial biology such as autophagy, structure, biosynthesis, and function.

Suitable oxidation of mitochondria plays a major role in energy metabolism. However, excessive ROS produced during mitochondrial dysfunction is associated with the development of NAFLD and obesity [1]. This pathological mechanism is associated with changes in NF-κB and PGC-1α activities [19, 20]. Furthermore, redundant ROS is responsible for cell apoptosis [21]. This study also proved that ROS accumulation in hepatocyte cell lines treated with OA or with a SIRT-1 inhibitor decreases cell viability and induces apoptosis. The activation of SIRT-1 prior to OA stimulation prevents this injurious effect. These results suggest that SIRT-1 is involved in the regulation of cell viability, apoptosis, and mitochondrial ROS.

Autophagy refers to the degradation of non-specific cytoplasmic proteins, organelles, and other substrates, and represents a mechanism of cell death regulation, different from apoptosis and pyroptosis. Autophagy resists cell pressure and promotes cell viability [22], while excessive autophagy adversely causes cell death. The gold standard markers to assess autophagy pathways are LC3B, Beclin-1, and p62. Especially, LC3B, an early marker of autophagy [20], induces autophagosome membrane expansion and fusion [22]. Besides, Beclin-1 promotes the initiation of autophagy by mediating the formation of autophagosomes. However, p62 aggregates outside the lysosomes and is involved in selective autophagy [23], in which the first steps of autophagy are impaired [24]. This knowledge was used to speculate on the trend of autophagy in cells, by detecting the changes in p62, Beclin-1, and LC3B protein expression. Experiments in mice showed that HFD increased the expression of p62 and reduced that of Beclin-1. These changes led to decreased autophagy, were not conducive to homeostatic liver lipid metabolism, and resulted in liver lipid accumulation, visible as numerous lipid droplets in liver tissues. Moreover, the same changes in p62 and Beclin-1 protein expression occurred in OA-stimulated cells. The ultrastructure analyses showed few autophagosomes. Additionally, lipid droplets were present in the intracellular space.

Moderate mitochondrial autophagy plays a crucial role in mitochondrial homeostasis by removing damaged mitochondria and contributing to cell metabolism. Abnormal mitochondria lead to imbalanced mitochondrial homeostasis and induction of mitochondrial autophagy. When mitochondria undergo autophagy, they usually show multilayered structures. In vitro, the present findings showed that excess of fat induced oil particle accumulation in the hepatocytes, and resulted in mitochondrial swelling, and even fracture. Second, the expression of the fission protein MFF diminished significantly, but that of the fusion protein MFN1 increased, both phenomena inducing abnormal mitochondrial structure. Putative mechanisms behind these changes were the increase of anomalous mitochondria to adapt to the over-accumulation of lipids. Moreover, the activation of SIRT-1 effectively promoted mitochondrial autophagy, which plays an important role in maintaining a dynamic balance in mitochondria.

PGC-1α is a marker of mitochondrial biogenesis, and as a transcriptional coactivator that enhances fatty acids oxidation and glycogenesis [25], it improves intracellular energy utilization [26]. The stability of PGC-1α is related to mitochondrial content [27, 28]. The lack of PGC-1α reduces the reversion of mitochondria, increases defective mitochondria, and eventually leads to cell death [29]. In addition, SIRT-1/PGC-1α have been shown to be part of a network regulating mitochondrial biogenesis in skeletal muscles [30]. In this regard, the changes operated by SIRT-1 on PGC-1α activity are consistent with previous reports [30, 31]. The reduction of SIRT-1 has various adverse effects on cells, such as decrease of viability, or defective mitochondrial structure and metabolism.

Mitochondrial fusion and fission regulate mitochondrial homeostasis, quantity, and even function [32]. Disrupted mitochondrial fission/fusion reduce energy production, increase oxidative stress, and even cell death [3, 32]. Mitochondrial fusion is mainly regulated by MFN1 (fusion protein, a dynamic-associated GTPase), which fuses the outer membranes of mitochondria [3]. MFF plays a role in mitochondrial fission. The decrease of SIRT-1 directly lowered mitochondrial fission by reducing MFF protein levels. This was reversed by SIRT-1 activation. OA treatment increased mitochondrial fusion, and decreased fission. Taken together, the analysis of these phenotypes indicates that mitochondria may become larger by a compensatory fusion to adapt to excessive lipid metabolism induced by OA.

It has been reported that hindered mitochondrial dynamics (fusion or fission) can lead to increased levels of some triacylglycerols, while autophagy can reverse this effect [33]. Together with this report, the present data indicate that lipid excess reduces SIRT-1, and that lipophages are not activated to a corresponding degree to degrade excessive lipids. This lack of regulation eventually leads to lipid accumulation and cell lipotoxicity.

Disruption of mitochondrial membranes, increase of ROS, fragmentation of mitochondria, and abnormal mitochondrial structure all promoted the initiation of mitochondrial autophagy [34]. The present findings demonstrate that excess of lipids and low expression of SIRT-1 lead to PGC-1α imbalance, and result in disordered mitochondrial biosynthesis and excess of ROS. Ultimately, mitochondrial autophagy is initiated.

### Study strengths and limitations

In the present study, there were several strengths. First, although mitochondrial disease was considered as the essence of NAFLD, few studies have performed the regulatory relationship between SIRT-1/PGC-1a and mitochondrial biology in the disease. Second, to further explore whether mitochondrial and lipid autophagy were regulated by SIRT-1, the molecular mechanism and subcellular structures influenced by SIRT-1 were included in this study. However, there are some limitations in this paper. The SIRT-1 related mechanisms were solely verified in vitro experiments. Therefore, in vivo studies are required to verify the finding.

## Conclusion

In conclusion, excess of fat downregulates SIRT-1/PGC-1α pathway and disturbs mitochondria’s biological activity, damaging them. It also decreases lipophages, causing lipid accumulation in cells and toxicity that ultimately leads to cell apoptosis. These results may point SIRT-1/PGC-1α pathway as a good target for further exploration of adjuvant therapy in NAFLD treatment.

## Data Availability

Not applicable.

## Abbreviations

NAFLD: Nonalcoholic fatty liver disease
HFD: High-fat-diet
OA: Oleic acid
ROS: Reactive oxygen species
TC: Total cholesterol
TG: Triglycerides
AST: Aspartate aminotransferase
ALT: Alanine aminotransferase
MDA: Malondialdehyde
SOD: Superoxide dismutase
SIRT-1: Sirtuin 1
PGC-1ɑ: Peroxisome proliferator-activated receptor-gamma coactivator -1a
COX-IV: Cytochrome c oxidase IV
p62: p62/SQSTM1, sequestosome 1
PPAR-ɑ: Peroxisome proliferator-activated receptor-ɑ
MFF: Mitochondrial fission factor
MFN1: Mitofusin-1
LC3: Light chain 3
LC3B: Light chain 3B
MA: Mitochondrial autophagy
PG: Phagophores
va: Vacuole
LD: Lipid drops
SM: Swollen and ruptured mitochondria
ASS: Autolysosomes
LA: Lipophagy
T6: Tenovin-6
CAY: CAY-10602
